# Imported PER-1 producing *Pseudomonas aeruginosa*, PER-1 producing *Acinetobacter baumanii *and VIM-2-producing *Pseudomonas aeruginosa *strains in Hungary

**DOI:** 10.1186/1476-0711-7-12

**Published:** 2008-05-30

**Authors:** Dora Szabó, Julia Szentandrássy, Zsuzsa Juhász, Katalin Katona, Károly Nagy, László Rókusz

**Affiliations:** 1 Institute of Medical Microbiology, Semmelweis University, Budapest, H-1089, Nagyvárad tér 4., Hungary; 2 Department of Microbiology, State Health Center, Budapest, H-1134 Róbert Károly krt. 44., Hungary; 3 Burn Unit, State Health Center, Budapest, H-1134 Róbert Károly krt. 44., Hungary; 4 First Department of Medicine, State Health Center, Budapest, H-1134 Róbert Károly krt. 44., Hungary

## Abstract

**Introduction:**

*Pseudomonas aeruginosa *and *Acinetobacter baumanii *are important nosocomial pathogens with wide intrinsic resistance. However, due to the dissemination of the acquired resistance mechanisms, such as extended-spectrum beta-lactamase (ESBL) and metallo beta-lactamase (MBL) production, multidrug resistant strains have been isolated more often.

**Case presentation:**

We report a case of a Hungarian tourist, who was initially hospitalized in Egypt and later transferred to Hungary. On the day of admission PER-1-producing *P. aeruginosa*, PER-1 producing *A. baumannii*, SHV-5-producing *Klebsiella pneumoniae *and VIM-2-producing *P. aeruginosa *isolates were subcultured from the patient's samples in Hungary. Comparing the pulsed-field gel electrophoresis (PFGE) patterns of the *P. aeruginosa *strains from the patient to the *P. aeruginosa *strains occurring in this hospital, we can state that the PER-1-producing *P. aeruginosa *and VIM-2-producing *P. aeruginosa *had external origin.

**Conclusion:**

This is the first report of PER-1-producing *P. aeruginosa*,and PER-1-producing *A. baumanii s*trains in Hungary. This case highlights the importance of spreading of the beta-lactamase-mediated resistance mechanisms between countries and continents, showing the importance of careful screening and the isolation of patients arriving from a different country.

## Introduction

*Pseudomonas aeruginosa *and *Acinetobacter baumanii *are very important nosocomial pathogens mainly in intensive care units, being responsible for various types of infections with more and more limited therapeutic options [1].

The *P. aeruginosa *have significant intrinsic resistance to antibiotics [2]. Therefore, the antipseudomonal beta-lactams such as ticarcillin, piperacillin, ceftazidime, cefepime, aztreonam, and the carbapenems have an important therapeutic value. Three mechanisms of beta-lactam resistance are predominant: production of beta-lactamases, loss of outer membrane proteins and up-regulation of efflux pumps. Most strains of *P. aeruginosa *which are resistant to third-generation cephalosporins produce a chromosomally mediated molecular class C beta-lactamase, the AmpC enzyme [2]. However, acquired beta-lactamases encoded by mobile genetic elements are important resistance mechanism in *P. aeruginosa *and in *Acinetobacter *spp. as well. Acquired resistance to beta-lactams can lead to therapeutic failure, especially when it is associated with resistance to other classes of drugs, such as aminoglycosides and fluoroquinolones. Among these enzymes acquired, PER (*Pseudomonas *extended resistance), a class A extended-spectrum beta-lactamase (ESBL), occurring less frequently has clinical importance by conferring resistance to oxyimino beta-lactams [3]. Poor outcome as a result of infection caused by PER-1 producers has been reported [4]. The also acquired metallo beta-lactamases (MBLs) of molecular class B can cause resistance to all beta-lactams except monobactam. The genes of MBLs are usually part of class I integron together with gene cassettes encoding resistance genes. Several types of MBL enzymes have been identified in *P. aeruginosa *among which the VIM-type enzymes appear to be the most prevalent [5].

Here we report the first detection of PER-producing *P. aeruginosa*, *A. baumannii *isolates and VIM-producing *P. aeruginosa *in Hungary from a patient, who was hospitalized in Egypt and transferred to Hungary. This work illustrates the dissemination of bacteria carrying PER-type ESBL and VIM-type MBL enzymes.

## Case presentation

In April, 2006 a 53-year-old Hungarian tourist was involved in a severe terror-attack in Egypt. He was initially hospitalized because of his burn, mechanical injuries and sepsis syndrome in Egypt. Five days later he was transferred in comatose, hypoxic and hypothermic condition to the Burn Unit of State Health Center, Budapest, Hungary. On the day of admission, bacterial cultures taken from burn wound. Based on the different colony morphology and antibiotic susceptibility patterns three different *P. aeruginosa *strains – an ESBL-producing *P. aeruginosa *(PA1), an imipenem-resistant *P. aeruginosa *(PA2), an MBL-producing *P. aeruignosa *(PA3), were observed and furthermore ESBL-producing *Klebsiella pneumoniae *(ESBL-KP), methicillin-resistant *Staphylococcus aureus *(MRSA) and *Enterococcus faecalis *(EF) were isolated. Next day ESBL-producing *A. baumanii *(ESBL-AB) and PA2 were cultured from the patient's canul. During his hospitalization from his nose PA1, ESBL-AB, ESBL-KP, MRSA, from his throat PA1, ESBL-AB, ESBL-KP, from his trachea PA3, ESBL-AB, ESBL.KP,-MRSA and from his wound PA1, PA3, ESBL-AB, ESBL-KP, MRSA and EF were isolated. Blood cultures were taken nine occasions and PA1, ESBL-KP, MRSA and EF were subcultured. The patient received adequate supportive treatment and empirically cefepime, subsequently meropenem and vancomycin were administered intravenously at high dosage. The aminoglycosides were synergy resistant. On the 8^th ^day of the hospitalization in Hungary the patient died.

The isolates were identified by VITEK 2 (BioMérieux, Marcy l'Etoile, France). On a routine antibiogram synergy was observed between amoxycillin/clavulanic acid and cefepime or ceftazidime disks **(**Oxoid, Basingstoke, Hampshire, United Kingdom) in *K. pneumoniae *and in *A. baumanii *strains but no synergy was observed in *P. aeruginosa *strains. The minimum inhibitory concentrations (MICs) of the antimicrobial agents were determined by E-test (AB Biodisk, Solna, Sweden) and by using the interpretative criteria of the Clinical and Laboratory Standards Institute [6]. Pulsed-field gel electrophoresis (PFGE) of the *SpeI*-digested (New England Biolab, Beverly, MA) genomic DNA of *P. aeruginosa *was performed and analyzed as described previously [7]. Isoelectric focusing (IEF) was performed, as described previously [8]. In the PCR assays primers specific to *bla*_TEM _[9], *bla*_SHV _[9], *bla*_PER _[10], *bla*_OXAs _[11], *bla*_IMP_, *bla*_VIM _and *bla*_classI _genes [12] were used. The nucleotide sequences were determined using the ABI 3100 Genetic Analyzers (Applied Biosystems, Foster City, CA). Sequences were analyzed using NCBI BLAST search.

Three different antibiotic susceptibility patterns were observed in *P. aeruginosa *strains isolated from the patient on the day of admission (Table 1). All of them were resistant to cephalosporins. The PA1 *P. aeruginosa *strain was sensitive only to carbapenems, the other *P. aeruginosa *strain (PA2) was sensitive only to meropenem and both PA1 and PA2 were resistant to all the other tested antibiotics. The third *P. aeruginosa *(PA3) strain was sensitive to aztreonam and netilmicin and resistant to the all cephalosporins and carbepenems. However, the imipenem MIC value decreased in the presence of EDTA from > 256 to 12 μg/ml suggesting the presence of an MBL enzyme. The *A. baumanii *isolate was resistant to all cephalosporins and aztreonam, but the ceftazidime and cefotaxime MIC values decreased in the presence of clavulanic acid suggesting the presence of an ESBL enzyme. The *K. pneumoniae *strain had also ESBL phenotype based on the MIC values and it was sensitive to carbapenems, amikacin and ciprofloxacin as well.

**Table 1:** The antibiotic susceptibility of *P. aeruginosa*, *A. baumanii *and *K. pneumoniae *strains.

Antimicrobial agents	MIC values (μg/ml)				
	
	*P. aeruginosa *(PA1)	*P. aeruginosa *(PA2)	*P. aeruginosa *(PA3)	*A. baumanii*	*K. pneumoniae*
Ceftazidime	> 256	> 256	> 256	> 256	> 256
Ceftazidime/clavulanic acid	> 4	> 4	> 4	< 0.64	3
Cefotaxime	> 32	> 32	> 32	> 32	> 32
Cefotaxime/clavulanic acid	> 1	> 1	> 1	0.23	0.38
Ceftriaxone	> 256	> 256	> 256	> 256	> 256
Piperacillin/tazobactam	> 256	> 256	> 256	> 256	> 256
Cefepime	> 32	> 32	> 32	> 32	> 32
Aztreonam	> 64	32	8	> 64	> 64
Imipenem	2	32	> 256	1	1
Imipenem+EDTA	1.5	32	12	1.5	< 1
Meropenem	0.25	1	> 32	0.38	0.25
Amikacin	> 256	6	> 256	48	1.5
Gentamicin	16	> 256	> 256	32	> 256
Netilmicin	> 32	> 32	2	1	> 32
Tobramycin	> 256	> 256	32	32	24
Ciprofloxacin	> 32	> 32	> 32	> 32	1

The PCR and sequencing results are summarized in Table 2. TEM-1 genes were identified in *A. baumanii *and *K. pneumoniae*. OXA genes were recognized in PA1 and PA3 strains. PER-1 genes were found in PA1 and *A. baumanii *isolates and SHV-5 was found in *K. pneumoniae*. Only the PA3 isolate harbored the VIM-2 gene. The OXA-10 and VIM-2 genes were located on the same class I integrons, as the sequencing result of class I integron showed.

**Table 2:** The characteristics of the isolated Gram-negative strains.

	TEM PCR	SHV PCR	PER PCR	OXA PCR	VIM PCR	PFGE	pI(s)
*P. aeruginosa *(PA1)	negative	negative	PER-1	OXA-10	negative	A type	5.3, 6.1, 8.0
*P. aeruginosa *(PA2)	negative	negative	negative	negative	negative	B type	8.0
*P. aeruginosa *(PA3)	negative	negative	negative	OXA-1	VIM-2	C type	5.3, 7.4, 8.0
*A. baumanii*	TEM-1	negative	PER-1	negative	negative	ND	5.3, 5.4, > 8.5
*K. pneumoniae*	TEM-1	SHV-5	negative	negative	negative	ND	5.4, 8.2

ND: not determined

According to IEF test results, all the isolates contained β-lactamases. Isoelectric focusing confirmed the expression of PER-1 (pI 5.3), TEM-1 (pI 5.4), OXA-1 (pI 7.4), OXA-10 (pI 6.1), SHV-5 (pI 8.2), VIM-2 (pI 5.3) and the chromosomal AmpC cephalosporinase (pI 8 or > 8.5) enzymes in the different strains (Table 2).

The three *P. aeruginosa *isolates, PA1, PA2 and PA3, showed three different PFGE patterns (data not presented) suggesting that they belonged to different clones (Table 2). Comparing these PFGE patterns to six other *P. aeruginosa *strains occurring in this Hungarian hospital during the same period, no similarity was found suggesting the external origin of PA1, PA2 and PA3.

## Discussion

Based on the antibiotic susceptibilities, IEF and the sequencing result we can state that the PA1 strain and the *A. baumanii *strain produced PER-1 ESBL enzyme. The *A. baumanii *strain also produced TEM-1 and the PA1 strain OXA-10 broad-spectrum β-lactamase. The PA2 strain produced only chromosomal AmpC cephalosporinase as suggesting that loss of OprD was responsible for the imipenem resistance. The PA3 strain produced the OXA-1 broad-spectrum β-lactamase and the VIM-2 MBL enzyme. The *K. pneumoniae *strain produced SHV-5 and TEM-1 enzyme.

According to the type of ESBL enzyme produced by different bacteria species SHV- TEM- and CTX-M type ESBL-producing *Klebsiella *spp. and *Escherichia coli *strains have been isolated in Hungary so far [13,14] – while in Egypt the ESBL phenotype has been observed in *K. pneumoniae *and CTX-M-type ESBL in *E. coli *strains [15,16]. The PER-1 beta-lactamase has been considered to be significant only in Turkey for years [17]. However, the PER-1 beta-lactamase found mainly in *P*. *aeruginosa *has been detected in many countries such as Turkey, France, Belgium, Spain, Italy, Poland, Romania, Japan and South Korea until now [10,18-25]. The PER-1 production in *Acinetobacter *spp. was observed more often in Turkey and in Korea [4,17,26,27]. According to our knowledge this is the first report of PER-1-producing *P. aeruginosa *and PER-1-producing *A. baumanii *in Hungary and since that other strains of PER-1 producing *P. aeruginosa *have been isolated [28].

Several types of MBL enzymes – IMP-type, VIM-type, SPM-1, GIM-1, SIM-1 – have been identified in *P. aeruginosa *[5]. The VIM enzymes are the most common in Europe [29]. The VIM-2 enzyme detected in this case has been previously found in several species worldwide and it seems to be the most prevalent allelic form [30-35]. In Hungary VIM-producing *P. aeruginosa *strains have been isolated but in Egypt MBL-producing strains have not been reported until now [36,37].

Furthermore the result of the PFGE analysis of the PA1, PA2 and PA3 confirms, that the three *P. aeruginosa *strains – the PER-1 producing *P. aeruginosa *(PA1), the OprD-loss *P. aeruginosa *(PA2) and VIM-2-producing *P. aeruginosa *(PA3) strains – had external origin and could be transferred from Egypt to Hungary. The PER-1 producing *P. aeruginosa *and *A. baumanii *strains disappeared from the hospital, no more infections have been detected with these strains since then.

The emergence and subsequently spread of PER-producing and MBL-producing strains are alarming. Supposing, that these resistance mechanisms might also exist in other countries the dissemination of PER-enzyme could be more prevalent over the world, particularly due to the unsolved problem of routine screening for ESBL-production in *P. aeruginosa *strains. The CLSI recommendation for screening the ESBL-production in different bacteria species is uncomplete, exist just for *Klebsiella *spp. and *Escherichia coli *strains [6].

In conclusion, this work confirms the emergence of PER-1-producing *P. aeruginosa *and *A. baumannii *isolate, VIM-2 producing *P. aeruginosa *strains in Hungary. Furthermore it illustrates the possibility of the inter-country and the inter-continent spread of the beta-lactamase-mediated resistance mechanisms. Our study features the intercontinental spread of antimicrobial resistance, showing the importance of careful screening and the isolation patients arriving from a different country.

## Competing interests

The authors declare that they have no competing interests.

## Authors' contributions

LR and ZJ provided clinical care, literature search and edited the manuscript, JS and KK identified the bacteria, performed the antibiotic susceptibility tests, DS carried out the molecular studies, characterized the bacteria and drafted the manuscript, NK drafted the manuscript. All authors read and approved the final manuscript.

## Consent

Written informed consent was obtained from the patient's relative for publication of this case report. A copy of the written consent is available for review by the Editor-in-Chief of this journal.
